# Predicting Liquid Crystal Behavior with Artificial Neural Networks

**DOI:** 10.3390/mi16121392

**Published:** 2025-12-09

**Authors:** Sarah Chattha, Simant R. Upreti, Philip K. Chan

**Affiliations:** Department of Chemical Engineering, Toronto Metropolitan University, 350 Victoria Street, Toronto, ON M5B 2K3, Canada; sarah.chattha@torontomu.ca (S.C.); supreti@torontomu.ca (S.R.U.)

**Keywords:** LCs, artificial neural networks, artificial intelligence

## Abstract

Liquid crystals (LCs) with fluid-like flow and solid-like molecular orientation find important applications in optical display and sensor technologies. Predicting the mean steady-state polar angle and refractive index is crucial for optimizing LC performance. While conventional predictive models such as those based on continuum theories require complex and computationally intensive numerical simulations, this study employs artificial neural networks (ANNs). In particular, they are developed to predict the mean steady state polar angle and refractive index from surface viscosity and anchoring energy. Using the train, validation, test method, ANN_A4 (R^2^ = 0.9995) and ANN_B2 (R^2^ = 0.9969) are found to have the highest predictive accuracy. On the other hand, using the K-Fold cross-validation, the results significantly differ, with the best performance shown in ANN_A5* (R^2^ = 0.40767) and ANN_B4* (R^2^ = 0.93799). Coupled with the low latency of ANNs, these results indicate that ANNs have significant potential in LC modeling, especially for use in the computationally intensive optimization of LC-based technologies.

## 1. Introduction

**Figure 1:**
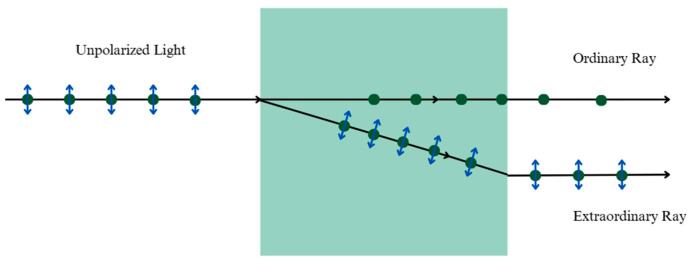
Schematic depiction of birefringence. The ordinary ray has polarization in a direction perpendicular to the optical axis of the material, and obeys the law of refraction. The extraordinary ray has polarization in the direction of the optical axis of the LC. These two rays may travel at different speeds and with different polarizations, resulting in various optical effects [2].

Liquid crystals (LCs) are a unique state of matter that exhibits properties between those of conventional liquids and solid crystals. Unlike simple fluids, their molecules maintain a degree of orientational order, giving rise to anisotropic physical properties that can be controlled by external factors such as temperature, electric fields, and surface interactions [1,2]. This anisotropy shown in Figure 1 allows for precise control over light transmission, polarization, and phase retardation based on the stimuli encountered allowing LCs to be indispensable for modern technologies [3,4,5,6,7,8,9,10,11,12,13,14,15].

In thin film configurations, LCs display behaviors distinct from their bulk counterparts due to the influence of reduced dimensionality, and surface interactions. These unique properties have propelled the use of thin LC films in advanced display technologies.

Thin film LC sensors have found applications in medical diagnostics [3,4,5,6,7], environmental monitoring [8,9,10,11], and food safety [12,13,14]. Emerging research is focusing on integrating LC thin films with microfluidic platforms and wearable devices, enabling portable and multifunctional sensing solutions [15,16]. Furthermore, advancements in nanotechnology and surface chemistry are expected to enhance the sensitivity and selectivity of these sensors further.

**Figure 2:**
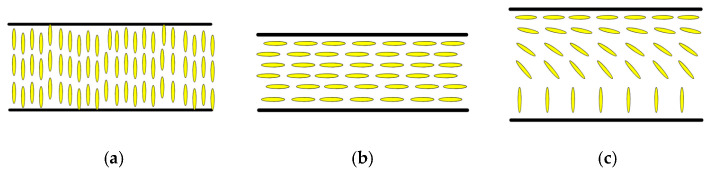
A schematic representation of typical surface alignments for nematic LCs upon an application of an external field consisting of (**a**) homeotropic for perpendicular arrangement of LCs along the interface, (**b**) planar alignment for parallel configuration along one specific plane, and (**c**) hybrid alignment in which the LCs align parallel to multiple planes within the same sample [2].

The performance of LC sensors is further significantly influenced by the anchoring conditions at the interfaces. Thin film sensors often use substrates treated with alignment layers to control the initial orientation of LC molecules with the different types of alignment shown in Figure 2. The interaction of analytes with the substrate or the LC itself alters these anchoring conditions, resulting in measurable shifts in molecular orientation. For example, biosensors based on LCs can detect the binding of biomolecules (e.g., proteins or DNA) through changes in surface anchoring energy, which induces observable texture changes in the film under polarizing optical microscopes (POMs) [3,4,5,6,7].

While LCs have been extensively studied for display applications, their behavior at confined interfaces remains an area of active research such as for the development of free energy formulas [17] and continuum models [18]. In particular, understanding the steady-state polar angle and the birefringence of LCs under varying surface viscosities and anchoring energies is crucial for optimizing their performance in emerging thin-film applications.

To unravel the complex behaviors of LCs and optimize their use in various applications, computational methods have played a crucial role in modeling and simulating LC systems. Among the most prominent approaches are molecular dynamics simulations (MD), Monte Carlo (MC) simulations, and continuum theories [19].

While traditional modeling approaches for LCs rely on the continuum theories such as Frank–Oseen and Ericksen–Leslie approaches [20], they often require complex modeling and intensive computational resources. As artificial intelligence (AI) continues to advance, machine learning methods, such as artificial neural networks (ANNs), present opportunities for LC modeling that is relatively simpler and computationally more efficient. In this regard, we aim to develop an ANNs capable of predicting the mean steady-state polar angle and the refractive index of LCs under different surface viscosities and anchoring energies. Utilizing data from prior studies, the ANNs are trained to recognize patterns in LC behavior and make accurate predictions.

Despite growing research on AI-driven LC sensors and predictive modeling, limited work has explored the application of ANNs in thin-film LC systems—particularly in predicting the reorientation of LC molecules under varying surface conditions. While previous studies have successfully implemented machine learning for applications such as biomedical diagnostics, gas sensing, and heavy metal ion detection, the prediction of steady-state director orientations and the effect of surface anchoring energy and surface viscosity in thin films remains underexplored. This gap presents an opportunity to leverage AI for improved predictive capabilities in LC dynamics, particularly under varying surface anchoring and viscosity conditions.

Compared to traditional numerical simulations, which can take several hours to days to compute a single scenario, the AI-based approach developed in this research significantly reduces computation time to just minutes while maintaining high predictive accuracy. Furthermore, implementing an ANN through MATLAB streamlines the modeling process by eliminating the need for multiple software tools typically used to derive and solve complex mathematical equations. For instance, previous work by Shadkami and Chan [21] employed the Galerkin finite element method with linear basis functions to solve a system of second-order differential equations to model LC orientation. In contrast, ANNs not only simplify the computational workflow but also remove the dependence on mesh-based methods, allowing for a more flexible and efficient approach to modeling LC behavior.

Beyond conventional liquid crystal systems, recent work [22,23,24] has expanded the investigation of anisotropic behavior into novel two-dimensional and nanoscale materials, offering new insight into how molecular ordering can be engineered. For example, graphene oxide-based liquid crystals show controllable birefringence and unexpected orientation behavior under shear [22] or confinement [23], demonstrating how external conditions can be used to tune optical anisotropy. Similarly, anisometric carbon quantum dots dispersed in LC hosts achieve field-responsive alignment and polarized emission [24]. These emerging material platforms reinforce the broader theme of this work: that manipulating orientational order, whether in traditional LCs or advanced hybrid systems, enables precise control of optical properties and opens pathways for next-generation photonic applications.

This work explores the application of artificial neural networks to predict the mean steady-state polar angle and effective refractive index, presenting a novel direction that can be further expanded to other liquid-crystal properties using both simulated and experimental datasets.

The remainder of this paper is organized as follows. Section 2 provides data processing details. Section 3 presents the ANN development. The results of deploying the ANNs are summarized in Section 4, and discussed in Section 5. Section 6 provides conclusions of this work.

## 2. Data Processing

This study utilizes ANNs to predict both the mean steady-state polar angle and the effective refractive index under varying surface viscosities and anchoring energies. The dataset used for training and validation was obtained from Shadkami and Chan [21], who employed the Frank–Oseen and Ericksen–Leslie theories to simulate LC performance at the aqueous interface. The surface anchoring energy was determined by the Rapini-Papoular expression, and the system’s distortion was described using the Euler–Lagrange equation to balance generalized and frictional forces.

Shadkami and Chan [21] set the initial conditions for the simulated data by confining the LC between Dirichlet boundary conditions, assuming strong homeotropic anchoring at the bottom glass surface in the absence of external fields. The governing equations and boundary conditions were nondimensionalized to aid in analyzing the system while reducing computational complexity.

A dataset consisting of 34,988 data points was generated for predicting the mean steady-state polar angle, while another dataset of 624 data points was used for predicting the effective refractive index. Both datasets covered a range of dimensionless surface viscosities (10 to 10,000) and anchoring energy values (1 to 100,000) at various dimensionless time steps. Each dataset was split into training (80%) and validation (20%) subsets to ensure robust model performance.

## 3. ANN Development

Two separate ANNs were developed—one to predict the mean steady-state polar angle and the other to predict the effective refractive index. The architectures varied in the number of hidden layers and neurons per layer to assess their effect on predictive accuracy. The computational resources used included a personal computer equipped with an Intel Core i7-11850H processor (8 cores, 16 logical processors) and 16 GB of RAM. The ANNs were trained and evaluated using MATLAB R2023a with the Deep Learning Toolbox, without GPU acceleration, as the computational load was manageable using CPU-based training.

### 3.1. Prediction of Mean Steady-State Polar Angle

To evaluate the impact of neural network complexity on prediction accuracy, we tested five different ANNs as listed in Table 1.

The input layer consisted of three dimensionless parameters: time step, surface viscosity, and surface anchoring energy. A single output node provided the predicted mean steady-state polar angle.

### 3.2. Prediction of Refractive Index

A separate ANN model was designed to predict the effective refractive index of the LC. Similarly to the input layer for the prediction of the mean steady state polar angle, the input layer consisted of surface viscosity, and surface anchoring energy. However, instead of the dimensionless time step, the dimensionless location along the thin film was taken as an input. Table 2 lists different ANN architectures tested to evaluate the neural network complexity and prediction accuracy.

### 3.3. Assumptions and Considerations

The methodology relies on several assumptions, including the generalizability of the ANN when trained on representative data. These data were generated by Shadkami and Chan [21] from the simulation of a rigorous theoretical model.

Some practical considerations include the heavy reliance on past data, necessitating computational resources for data generation as conducted by Shadkami and Chan [21]. It is worth noting that the ANN models are trained entirely on simulated data, so their predictions are reliable primarily within the range of the training dataset. Extrapolation beyond these limits is uncertain and should be interpreted with caution. While the models provide accurate predictions for the parameter ranges represented in the simulations, their performance on experimental data has not been validated in this study. Future work incorporating experimental measurements would be necessary to assess generalization and validate predictions for real LC systems.

### 3.4. Training and Optimization

Both ANN models were trained using the Levenberg–Marquardt (TrainLM) backpropagation algorithm, which is known for its fast convergence and efficiency, particularly with medium-sized datasets. The hidden layers used the “tansig” transfer function, the default in MATLAB’s Deep Learning Toolbox for feedforward neural networks [25]. For the prediction of the refractive index, the last layer employed the “ReLU” transfer function (referred to as “poslin” in MATLAB) to restrict outputs to non-negative values. Before training, the input data for polar angle prediction was normalized using MATLAB’s zscore function, ensuring features had zero mean and unit variance. For refractive index prediction with K-fold cross-validation, min–max normalization was applied to maintain physical bounds and aid convergence, especially after augmenting the dataset from 624 to 13,000 points. This augmentation increased the diversity of training examples, helping the network generalize more effectively.

To optimize training, key parameters were configured. The learning rate was set to 10^−3^, ensuring stable convergence while minimizing oscillations in the mean squared error (MSE) function. Training was allowed to run for a maximum of 1000 iterations (epochs), providing sufficient opportunities for learning without excessive computational cost. The process was designed to halt if either the performance goal of 10^−6^ was achieved, indicating an acceptably low MSE, or the maximum number of epochs was reached. Overfitting occurs when a model memorizes noise in the training data rather than learning underlying patterns, reducing generalization to unseen data. To mitigate this, a maximum of 100 validation failures was permitted before stopping training.

For K-fold cross-validation, the scaled conjugate gradient (TrainSCG) algorithm was used. This approach provided a more robust evaluation of model generalization by training and testing the network across multiple data splits, reducing the likelihood of overfitting due to lucky initializations. Model performance was evaluated using the coefficient of determination (R^2^) as the primary evaluator, ensuring alignment with the research objectives of accurately predicting both polar angles and refractive indices. Early stopping and monitoring of validation loss were applied consistently to prevent overfitting while maximizing predictive capability.

## 4. Results

This section presents the results of ANNs trained as described above to predict the mean steady-state polar angle (*θ*), and the effective refractive index of LCs under varying surface viscosities and anchoring energies. The models are evaluated based on their predictive accuracy, convergence behavior, and training efficiency while the performance of each ANN architecture is assessed using Mean Squared Error (MSE) and gradient behavior.

The following sections analyze the predictive performance of different ANN architectures, examining the impact of network depth, neuron count, and training dynamics on overall accuracy.

### 4.1. Prediction of Mean Steady-State Polar Angle Using TrainLM

For this purpose, we examined five ANNs, ANN_A1–ANN_A5, with different number of hidden layers and neurons per layer. Table 3 summarizes the training details.

#### 4.1.1. ANN_A1: 3 Hidden Layers, 10 Neurons

ANN_A1 exhibited moderate predictive accuracy (R^2^ = 0.8042), with training converging after 531 epochs. The model captured general trends but struggled with lower theta values, producing negative predictions. This suggests inadequate model complexity for learning intricate patterns, potentially due to insufficient neurons per layer.

**Figure 3:**
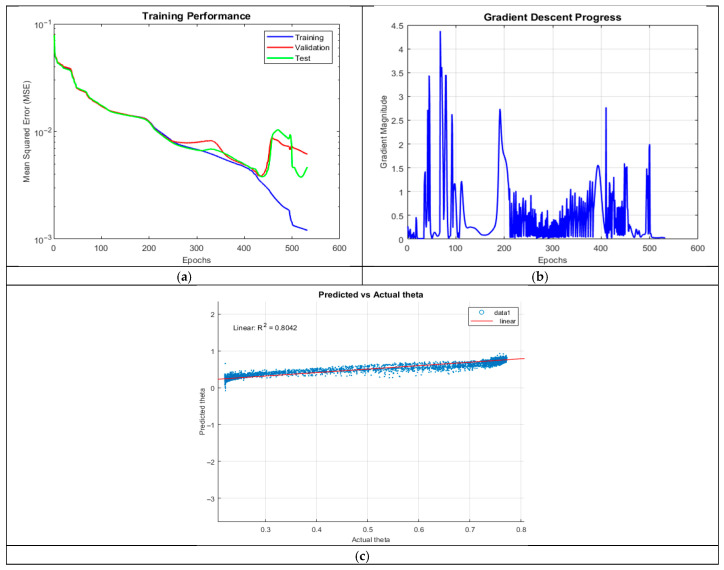
Training outputs of ANN_A1: (**a**) performance measuring the MSE; (**b**) magnitude of the gradient through training; and (**c**) comparison between the predicted and actual values.

The MSE plot in Figure 3 showed a clear divergence between training and validation MSE after 350 epochs, indicating the possibility of overfitting. Furthermore, the gradient plot revealed oscillatory behavior, with high initial spikes followed by a gradual decline near termination, suggesting instability in weight updates.

#### 4.1.2. ANN_A2: 3 Hidden Layers, 25 Neurons

Increasing the neuron count significantly improved performance (R^2^ = 0.9597). The network better captured nonlinearities, as reflected in the improved alignment between predicted and actual theta values.

**Figure 4:**
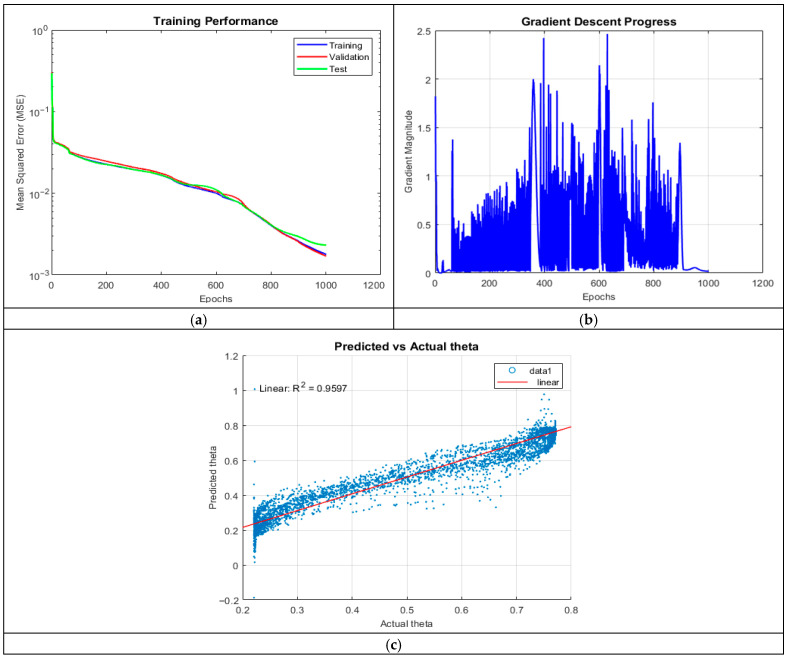
Training outputs of ANN_A2: (**a**) performance measuring the MSE; (**b**) magnitude of the gradient through training; and (**c**) comparison between the predicted and actual values.

The MSE plot in Figure 4 remained stable until 800 epochs, after which a slight divergence emerged, indicating overfitting. The presence of outliers at both smaller and larger theta values suggests sensitivity to certain input conditions, possibly due to non-uniform data distribution. Gradient oscillations persisted throughout training, with notable fluctuations up to the 850th epoch, indicating challenges in optimization stability.

#### 4.1.3. ANN_A3: 3 Hidden Layers, 50 Neurons

Despite a higher neuron count, ANN_A3 underperformed (R^2^ = 0.6574), with training manually halted after 12 h due to excessive computation time. The increase in complexity led to slower convergence, likely due to ineffective weight updates and poor gradient flow.

**Figure 5:**
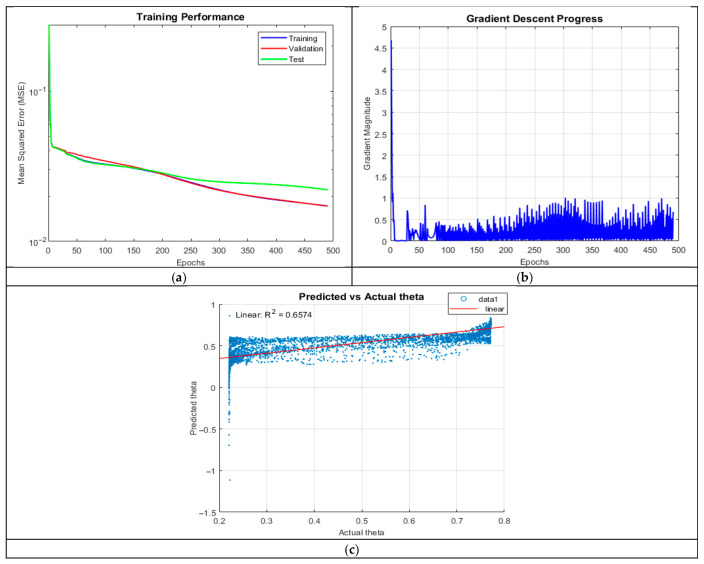
Training outputs of ANN_A3: (**a**) performance measuring the MSE; (**b**) magnitude of the gradient through training; and (**c**) comparison between the predicted and actual values.

The MSE plot in Figure 5 showed significant divergence between training and test curves, highlighting severe overfitting. The gradient plot revealed erratic oscillations, indicating unstable optimization. These results suggest that the network’s complexity exceeded the data’s representational capacity, leading to inefficient learning dynamics.

#### 4.1.4. ANN_A4: 5 Hidden Layers, 10 Neurons

Increasing network depth to five hidden layers yielded the best predictive accuracy (R^2^ = 0.9995). The prediction plot showed near-perfect alignment, with no significant outliers or negative predictions, demonstrating robust generalization.

**Figure 6:**
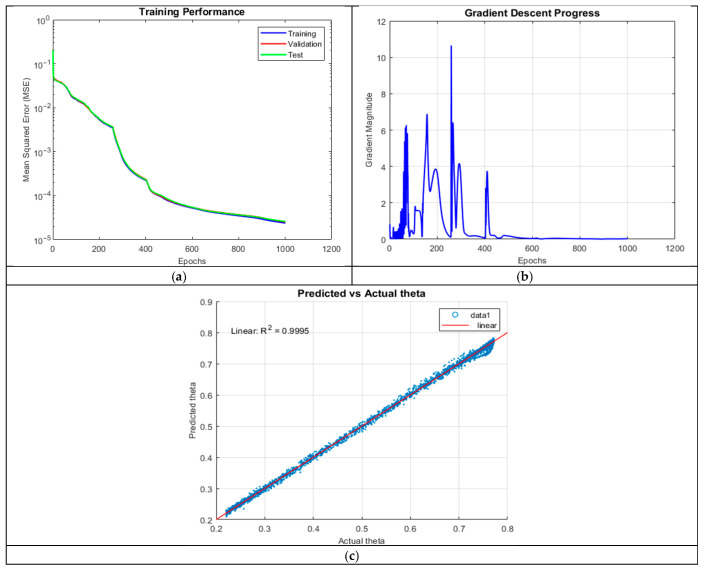
Training outputs of ANN_A4: (**a**) performance measuring the MSE; (**b**) magnitude of the gradient through training; and (**c**) comparison between the predicted and actual values.

The MSE plot in Figure 6 followed a smooth logarithmic decline, with almost no divergence between training, validation, and test sets, indicating excellent generalization. The gradient plot exhibited high initial magnitudes (exceeding 10), followed by stabilization after 450 epochs, suggesting efficient learning dynamics. These results highlight the advantages of deeper networks in capturing intricate physical dependencies, provided overfitting is controlled.

#### 4.1.5. ANN_A5: 5 Hidden Layers, 20 Neurons

ANN_A5 (R^2^ = 0.9993) showed a slight decline compared to ANN_A4. The model reached the 1000-epoch limit, and a single outlier at lower theta values indicated sensitivity to extreme cases. The slight increase in MSE suggests mild overfitting due to excessive parameterization, which may have reduced generalizability.

**Figure 7:**
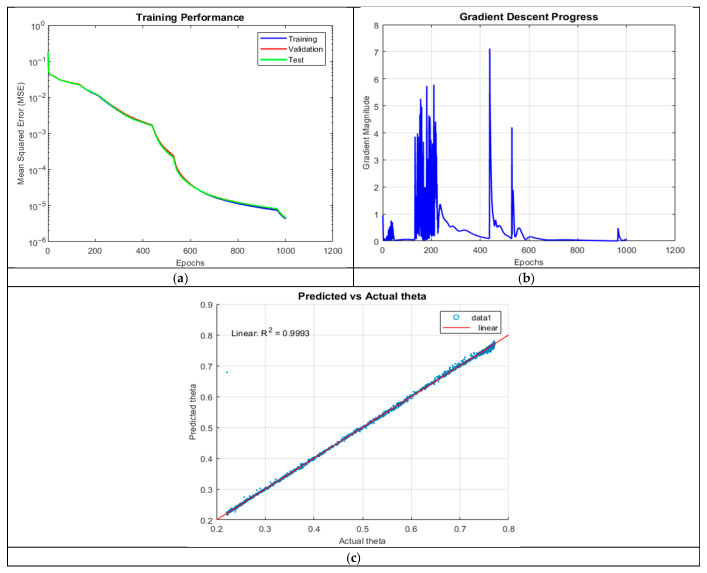
Training outputs of ANN_A5: (**a**) performance measuring the MSE; (**b**) magnitude of the gradient through training; and (**c**) comparison between the predicted and actual values.

The MSE plot in Figure 7 showed minimal divergence, but gradient oscillations were more pronounced than in ANN_A4, with sudden jumps at 410 and 520 epochs. These fluctuations suggest occasional instability in weight updates. While the model ultimately converged, the increased neuron count did not provide substantial improvements, highlighting the diminishing returns of additional complexity.

### 4.2. K-Fold Cross-Validation—Mean Steady-State Polar Angle Using TrainSCG

To further assess the robustness and generalization capability of the neural network developed for predicting the mean steady-state polar angle, a K-fold cross-validation procedure was performed. While the TrainLM-based model in Section 4.1 achieved a high accuracy for a single train/validation split, K-fold validation provides a more rigorous evaluation by repeatedly training the model on different subsets of the data. This procedure helps identify whether the high performance achieved with the architectures defined previously is consistently reproducible or dependent on the specific random partitioning of the dataset.

#### 4.2.1. Architecture and Training Set up

To further assess the generalizability of the networks evaluated in Section 4.1.1, the same architectures were trained and evaluated under a 5-fold cross-validation framework using the Scaled Conjugate Gradient (SCG) training algorithm. The Levenberg–Marquardt algorithm could not be used within the cross-validation framework due to instability during repeated training; specifically, several folds produced NaN outputs, indicating divergence of the Hessian approximation. TrainSCG is more stable for iterative retraining and is commonly recommended when LM becomes computationally expensive or unstable in repeated optimization cycles.

In all folds, the data were normalized using z-score normalization (as done previously), and the K-fold procedure was implemented. Performance was assessed for each fold shown in Table 4:

#### 4.2.2. Performance Overview

Overall, the networks demonstrated moderate and comparatively weak predictive performance, with R^2^ values ranging from 0.33 to 0.41, significantly lower than the near-perfect accuracy achieved using TrainLM in Section 4.1.1 (R^2^ ≈ 0.999). Although increasing the number of neurons improved performance modestly within the 3-layer networks, the gains were small, and deeper architectures did not consistently outperform shallower ones.

**Figure 8:**
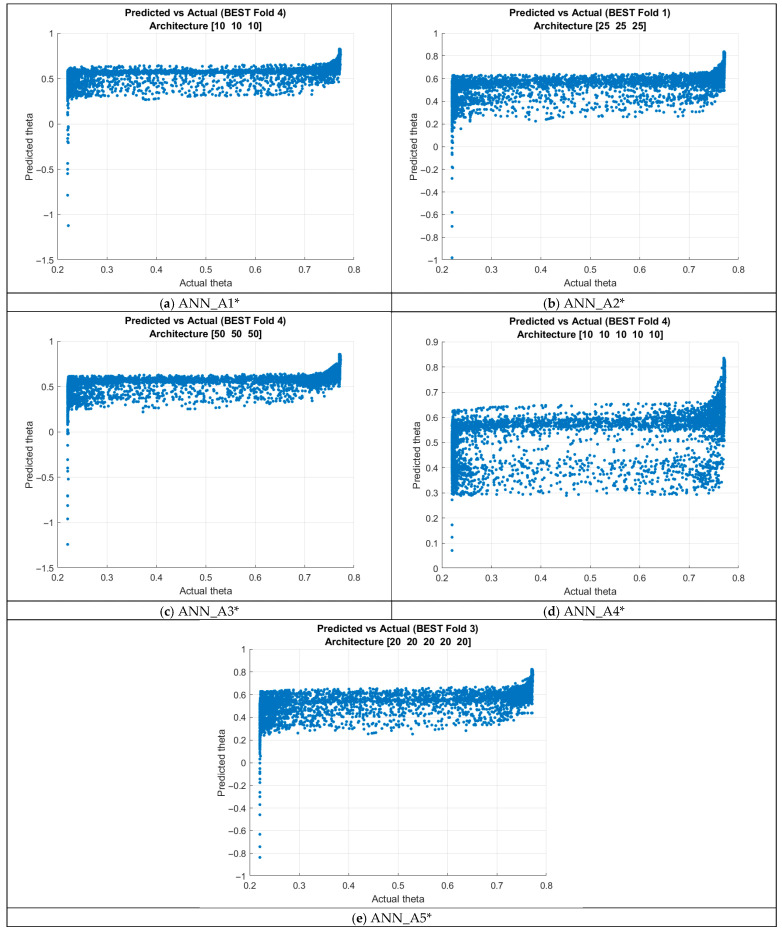
Training outputs using K-Fold cross-validation for ANN_A*.

The best-performing model, the ANN_A5* architecture, reached a mean R^2^ of 0.408, yet this still indicates limited predictive power and substantial deviation from ideal performance. The low standard deviations of RMSE and MAE across folds suggest that the results are consistent and not dominated by randomness; rather, they reflect a fundamental inability of SCG-trained networks to learn a highly accurate mapping under cross-validated conditions as shown in Figure 8.

The K-fold cross-validation experiments revealed a consistent decline in overall predictive performance compared to the TrainLM results presented earlier. While the TrainLM-based networks achieved near-perfect agreement with the target values (R^2^ ≈ 0.999), the SCG-trained networks produced considerably lower R^2^ values, ranging only from approximately 0.33 to 0.41 across all tested architectures. This discrepancy indicates that although the networks were capable of fitting the training data in a standard training–validation split, they did not generalize as effectively when subjected to a more rigorous and data-efficient evaluation framework.

Several factors likely contributed to this reduction in performance. First, the exceptionally high TrainLM results suggest that those networks may have been overfitting the training data, particularly given the deterministic nature of the simulated dataset. K-fold cross-validation reduces the amount of training data in each fold, making it more difficult for the networks to reproduce highly nonlinear features of the mapping, thereby exposing any reliance on fold-specific patterns learned during conventional training. Second, the SCG optimization algorithm is inherently less aggressive than TrainLM; it typically converges more slowly and may not fully minimize the loss for highly nonlinear mappings such as the steady-state polar angle. The reduced optimization strength becomes more pronounced when each fold contains fewer samples, limiting the model’s ability to achieve the same accuracy as in the full-dataset training scenario.

Additionally, increasing the number of neurons or hidden layers did not substantially improve performance under cross-validation. Although the 20-20-20-20-20 architecture exhibited the highest mean R^2^ (0.4077), the improvement was marginal and insufficient to compensate for the reduced training data per fold. This suggests that deeper architectures may be prone to underfitting in K-fold settings due to the higher number of parameters and reduced sample availability. Overall, the cross-validation experiments demonstrate that while the TrainLM networks achieved high accuracy, their generalization capability is limited. The K-fold SCG results emphasize the importance of cross-validated training and indicate that additional data or alternative regularization strategies may be required to develop more robust predictive models for *θ*.

While the TrainLM models achieved extremely high predictive accuracy (R^2^ ≈ 0.9995), the K-fold TrainSCG experiments revealed that the same architectures have limited generalizability when evaluated more rigorously. This discrepancy underscores the importance of cross-validated training and suggests that the high accuracy obtained from the original TrainLM networks may reflect overfitting rather than true predictive robustness.

### 4.3. Prediction of Refractive Index Using TrainLM

For this purpose, we examined six ANNs, ANN_B1–ANN_B6, with different number of hidden layers and neurons per layer. Table 5 summarizes the training details.

#### 4.3.1. ANN_B1: 2 Hidden Layers with 100 Neurons per Layer

The first ANN architecture, ANN_B1, consisted of two hidden layers, each with 100 neurons. The model achieved a coefficient of determination (R^2^) of 0.7751. The training process converged after 117 epochs, terminating upon satisfying the validation criteria. While this model captured general trends in the data, its limited depth may have constrained its capacity to learn complex feature representations. Shallow architectures often struggle to extract intricate patterns in highly nonlinear systems, which appears to be a limiting factor in this case.

The prediction accuracy plot for ANN_B1, which visualizes predicted refractive index values against actual values, reveals a systematic underestimation of larger refractive indices. This discrepancy suggests that the network fails to fully account for nonlinear variations in the underlying physical relationships governing refractive index behavior. Additionally, the scatter in predicted values indicates a lack of robustness, further reinforcing the fact that the network lacks sufficient complexity to generalize effectively.

**Figure 9:**
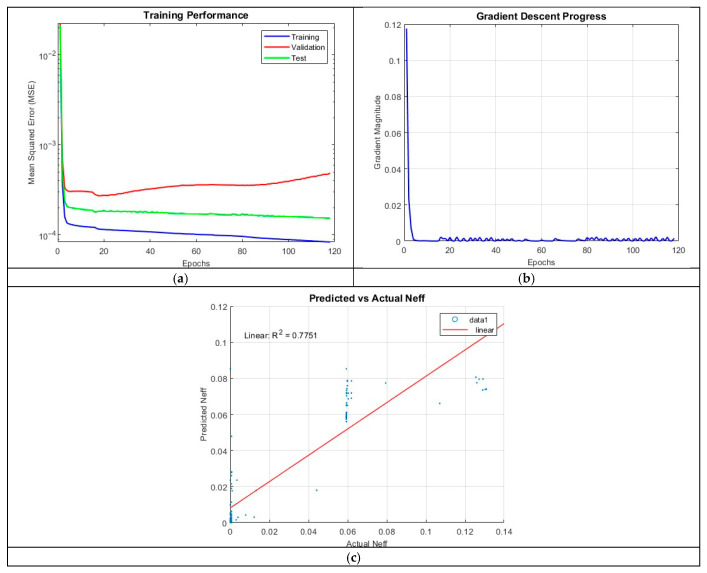
Training outputs of ANN_B1: (**a**) performance measuring the MSE; (**b**) magnitude of the gradient through training; (**c**) comparison between the predicted and actual values.

The MSE over epochs plot in Figure 9 shows a rapid decrease in error during the initial five epochs, reflecting efficient early-stage learning. However, beyond this point, the validation MSE begins to diverge from the training MSE, indicating overfitting. By the end of training, the final MSE values were 1.1589 × 10^−4^ (training), 2.7179 × 10^−4^ (validation), and 1.8384 × 10^−4^ (test). The gradient magnitude plot further supports this observation, showing a sharp initial decline followed by minimal subsequent updates. This behavior suggests that while the network initially adapts quickly, it reaches a local minimum where further optimization yields diminishing returns.

#### 4.3.2. ANN_B2: 3 Hidden Layers with 10 Neurons per Layer

ANN_B2 introduced an additional hidden layer while significantly reducing the number of neurons per layer to 10. This configuration resulted in a substantial improvement in performance, with the R^2^ increasing to 0.9969. Training concluded after 320 epochs, indicating a more extended learning process compared to ANN_B1. The improved performance suggests that increased network depth, even with fewer neurons per layer, enhances the model’s capacity to extract meaningful patterns.

**Figure 10:**
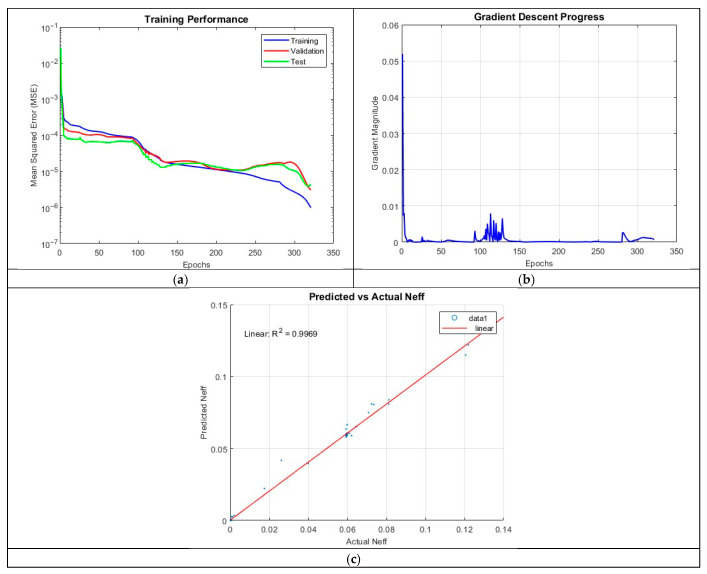
Training outputs of ANN_B2: (**a**) performance measuring the MSE; (**b**) magnitude of the gradient through training; (**c**) comparison between the predicted and actual values.

The prediction accuracy plot in Figure 10 for ANN_B2 shows a near perfect alignment between predicted and actual values. This result supports the notion that deeper networks, even with reduced neuron counts, provide better feature extraction and generalization. However, occasional mispredictions persist, suggesting that additional architectural refinements or regularization techniques may be necessary.

The MSE plot demonstrates a more stable training process, with validation and test errors closely following the training error. The final MSE values of 9.9053 × 10^−7^ (training), 3.0541 × 10^−6^ (validation), and 4.2544 × 10^−6^ (test) indicate that the model generalizes well, albeit with some degree of overfitting towards the later epochs. The gradient plot exhibits rapid early-stage learning, with gradients fluctuating around epoch 100 after which stabilization occurs, suggesting relatively efficient convergence.

#### 4.3.3. ANN_B3: 3 Hidden Layers with 25 Neurons per Layer

Maintaining three hidden layers but increasing the neuron count to 25 per layer in ANN_B3 resulted in a decline in predictive performance. The R^2^ value dropped to 0.9812, with training terminating after 287 epochs. This result indicates that simply increasing the number of neurons does not necessarily yield improved performance and may, in some cases, introduce additional training complexities.

**Figure 11:**
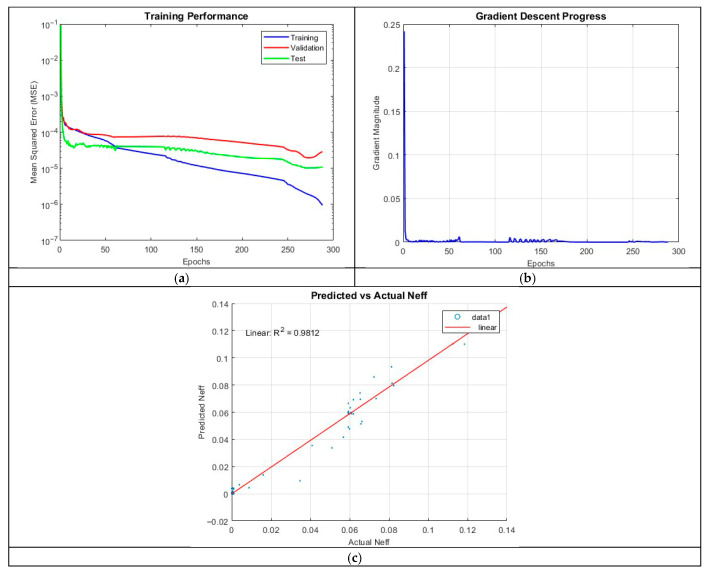
Training outputs of ANN_B3: (**a**) performance measuring the MSE; (**b**) magnitude of the gradient through training; (**c**) comparison between the predicted and actual values.

The MSE plot in Figure 11 further reveals a widening gap between training and validation errors, indicative of overfitting. The final MSE values were 1.8649 × 10^−6^ (training), 1.9549 × 10^−5^ (validation), and 1.0212 × 10^−5^ (test), reinforcing the observation that the model is less accurate than ANN_B2.

#### 4.3.4. ANN_B4: 5 Hidden Layers with 10 Neurons per Layer

Introducing additional depth in ANN_B4, which employed five hidden layers with 10 neurons per layer, led to a further decline in performance. The model achieved an R^2^ of 0.9209. Training was completed after 209 epochs, demonstrating that increasing the complexity of the neural network does not necessarily aid capturing the underlying physics governing the refractive index.

**Figure 12:**
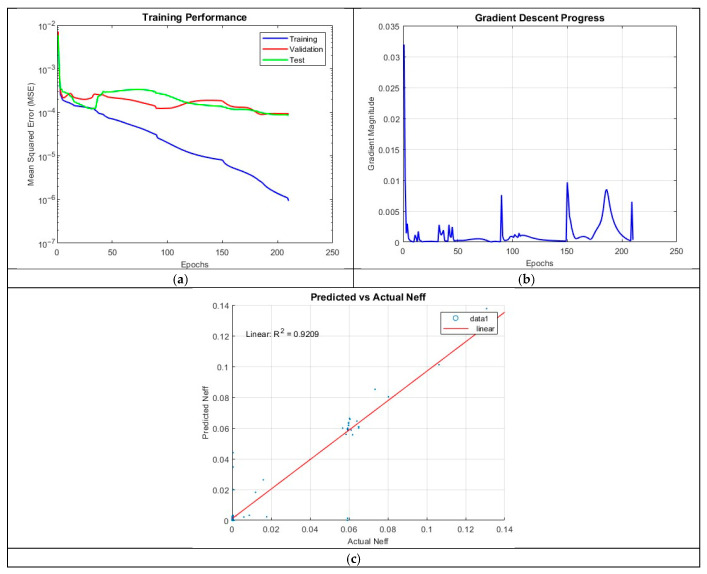
Training outputs of ANN_B4: (**a**) performance measuring the MSE; (**b**) magnitude of the gradient through training; (**c**) comparison between the predicted and actual values.

The prediction accuracy plot in Figure 12 for ANN_B4 shows agreement between predicted and actual values, particularly for larger refractive indices. However, this agreement was less than both ANN_B2 and ANN_B3. The MSE over epochs plot suggests a relatively well-behaved training process, with the final MSE values of 4.2546 × 10^−6^ (training), 8.9717 × 10^−5^ (validation), and 9.7719 × 10^−5^ (test) confirming good generalization.

#### 4.3.5. ANN_B5: 5 Hidden Layers with 20 Neurons per Layer

The architecture, ANN_B5, employed five hidden layers with 20 neurons per layer. This model achieved an R^2^ value of 0.9566 providing better results than ANN_B4. The training process terminated after 111 epochs, emphasizing the benefits of a deep network with a well-calibrated neuron count.

**Figure 13:**
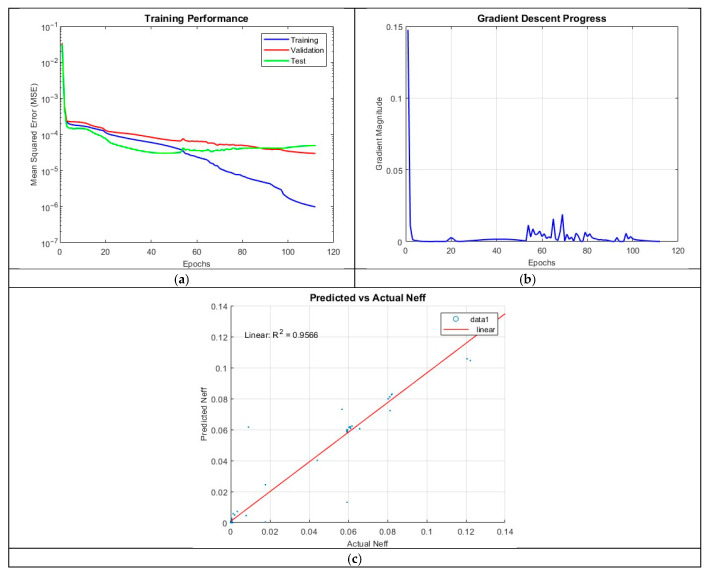
Training outputs of ANN_B5: (**a**) performance measuring the MSE; (**b**) magnitude of the gradient through training; (**c**) comparison between the predicted and actual values.

The prediction accuracy plot in Figure 13 demonstrates good alignment between predicted and actual values. The MSE plot reveals an efficient training process, with the final MSE values of 9.8595 × 10^−7^ (training), 2.9830 × 10^−5^ (validation), and 4.9577 × 10^−5^ (test) suggesting only minor overfitting. The gradient plot indicates occasional oscillations, likely due to minor instabilities in weight updates, but these do not significantly impact overall performance.

#### 4.3.6. ANN_B6: 5 Hidden Layers with 50 Neurons per Layer

Finally, ANN_B6 increased the neuron count to 50 per layer while maintaining five hidden layers. While the model performed well, achieving an R^2^ of 0.9568, its accuracy did not surpass ANN_B2 and ANN_B3. The addition of 30 neurons per layer from ANN_B5 did not significantly improve the performance of the neural network, resulting in a less efficient architecture compared to the other models.

**Figure 14:**
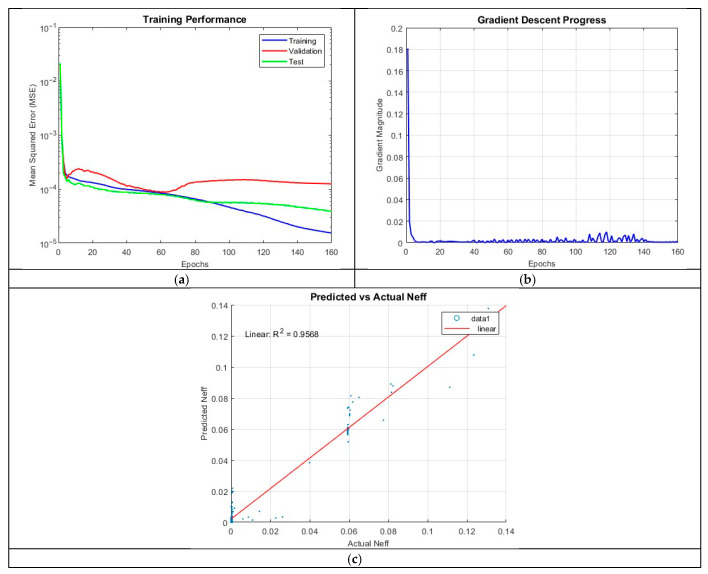
Training outputs of ANN_B6: (**a**) performance measuring the MSE; (**b**) magnitude of the gradient through training; (**c**) comparison between the predicted and actual values.

The final MSE values from Figure 14; 8.4324 × 10^−5^ (training), 8.7738 × 10^−5^ (validation), and 8.0418 × 10^−5^ (test) indicate poorer performance compared to ANN_B2, further reinforcing the conclusion that excessive neuron counts may lead to diminishing returns.

### 4.4. K-Fold Cross-Validation—Refractive Index Using TrainSCG

To further evaluate the predictive performance of ANNs for the effective refractive index, the same architectures tested with TrainLM were trained using a 5-fold cross-validation scheme and the scaled conjugate gradient algorithm. The original dataset of 624 points was augmented to 13,000 points using Latin Hypercube Sampling, ensuring uniform coverage of the input space and improving the network’s ability to generalize. Any negative refractive index values generated during augmentation were resampled to maintain physically meaningful predictions.

For this K-fold training, both the input variables and the output were normalized using robust min–max scaling rather than z-score normalization. This approach ensured that all features and the target were bounded between 0 and 1, reducing the likelihood of numerical instabilities and NaN predictions during SCG training.

The five-layer network with 10 neurons per layer (ANN_B4*) exhibited the best performance, achieving a mean R^2^ of 0.938, mean RMSE of 0.919, and mean MAE of 0.223. The predicted versus actual refractive index plot for this fold shows strong agreement across the full data range, indicating effective learning of nonlinear dependencies. Smaller architectures, such as the three-layer network with 10 neurons (ANN_B2*), also performed well with an R^2^ of 0.866, demonstrating that moderate network depth is sufficient for capturing the underlying physics. In contrast, excessively large networks—such as the two-layer 100-neuron network and the five-layer 50-neuron network—showed lower R^2^ values (0.700 and 0.721, respectively) despite stable training, suggesting that overparameterization can reduce generalization and increase sensitivity to fold partitions. Table 6 summarizes the findings below.

**Figure 15:**
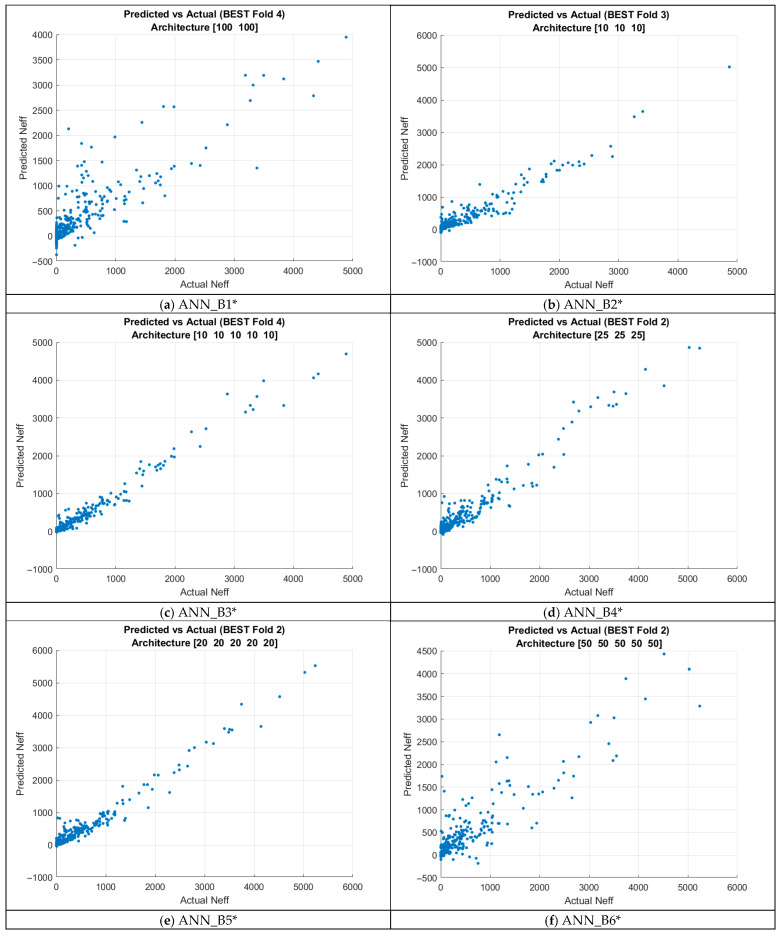
Training outputs using K-Fold cross-validation for ANN_B*.

Overall, the K-fold SCG results show that network performance improves with moderate depth and neuron count, while overly complex architectures may lead to decreased predictive accuracy. The use of augmented data allowed the networks to learn across a wider input space, and the resampling of negative refractive index values ensured physically consistent outputs. Figure 15 presents the predicted versus actual refractive index for the best fold of each architecture, illustrating the close alignment between predicted and measured values.

## 5. Discussion

For predicting the mean steady-state polar angle, ANN_A4 achieved the optimal balance of accuracy, stability, and generalization. Its deeper architecture facilitated hierarchical feature extraction, enabling precise predictions. Training was completed in about 3 min, positioning ANN_A4 as an efficient alternative to traditional computational methods based on continuum theory. The superior performance of deeper networks indicates that additional hidden layers effectively capture complex dependencies in input parameters. However, further optimization could enhance efficiency and mitigate overfitting risks. Techniques such as dropout, L2 regularization, or stricter early stopping could improve generalization by reducing reliance on specific neurons and halting training when validation loss plateaus. For models like ANN_A3, which exhibited excessive computational time and poor convergence, advanced optimization algorithms such as adaptive gradient clipping could stabilize weight updates and improve training efficiency. Additionally, Bayesian optimization for hyperparameter tuning could identify optimal architectures more effectively than manual trial-and-error. Outliers in predictions from ANN_A2 and ANN_A5 suggest sensitivity to specific input regions. A more balanced dataset with uniform distribution across the theta range could enhance robustness. Augmenting the training set with synthetic data generated through interpolation or perturbation could further improve predictive accuracy. Overall, while deeper networks generally enhance accuracy, optimal performance requires careful architectural tuning, regularization, and data augmentation. Refining these elements can make ANNs more precise and computationally efficient for predicting LC behavior.

When evaluating the same architectures using K-fold cross-validation with TrainSCG, predictive performance for the mean polar angle decreased substantially, with R^2^ values between 0.33 and 0.41. Despite using the same z-score normalization, the reduced effective training set per fold likely increased susceptibility to overfitting and underfitting. TrainSCG’s sensitivity to initial weights and convergence to suboptimal minima further contributed to lower performance. These K-fold results emphasize that while the TrainLM feedforward networks provide near-perfect predictions, smaller training splits and algorithmic differences reveal potential overfitting risks and the importance of robust validation strategies. Nonetheless, the K-fold evaluation offers valuable insight into model generalization and highlights limitations when data quantity is constrained.

For refractive index prediction, deeper networks generally improved accuracy when the number of neurons per layer was optimally selected. ANN_B2, with three hidden layers and 10 neurons each, achieved the highest accuracy (R^2^ = 0.9969), highlighting the importance of an optimized architecture for capturing nonlinear dependencies while maintaining efficiency. However, increasing the number of neurons per layer did not always improve performance. For example, ANN_B3, with 25 neurons per layer, suffered from overfitting, as evidenced by the growing disparity between training and validation errors. This outcome indicates that excessive neurons introduce redundancy, reducing optimization efficiency and generalization. Conversely, shallower models like ANN_B1, with two hidden layers of 100 neurons each, failed to capture the full complexity of refractive index dependencies. These findings align with deep learning research emphasizing the need for a balanced network depth and width.

In the K-fold scenario for the refractive index, the training dataset was augmented from 624 to 13,000 points, and min–max normalization was applied to maintain physical bounds and prevent negative predictions. Negative values were eliminated through iterative resampling, ensuring that all predictions remained physically meaningful. With these modifications, K-fold performance improved considerably, achieving R^2^ values between 0.90 and 0.94. This demonstrates that sufficient data quantity and appropriate normalization can mitigate convergence challenges inherent to TrainSCG, allowing the network to generalize effectively even with cross-validation splits. Notably, the best-performing K-fold architecture, ANN_B4*, differed from the TrainLM optimal network, highlighting how cross-validation can shift the preferred network choice toward models that better generalize to unseen data.

It is worth noting that the extremely high R^2^ values obtained from the train–validation–test split (e.g., ANN_A4 R^2^ = 0.9995) reflect performance on a single data partition, where the majority of data was used for training. Such results can be overly optimistic and may indicate overfitting rather than true predictive capability. To obtain a more realistic measure of model generalization, we performed K-fold cross-validation. These results yielded lower, more representative R^2^ values (ANN_A5* R^2^ ≈ 0.41 for polar angle prediction, ANN_B4* R^2^ ≈ 0.94 for refractive index prediction), demonstrating that while the networks capture underlying trends, their predictive performance is more modest across multiple data splits. The K-fold analysis, alongside metrics such as test RMSE and residual distributions, provides a robust evaluation of model reliability and emphasizes the importance of cross-validation when interpreting near-perfect fit metrics.

Across both polar angle and refractive index prediction tasks, network architecture significantly influenced performance. Deeper networks consistently outperformed shallower ones, provided the neuron count per layer was optimized. However, excessive neurons, as seen in ANN_A3 and ANN_B6, led to diminishing returns and potential overfitting. This underscores the importance of regularization to manage complexity. Computational cost is another critical factor. Larger networks required longer training times and faced convergence challenges without proper regularization. Extended training times could hinder practical deployment, particularly for real-time predictions where efficiency is critical. Conversely, overly simplistic models like ANN_A1 and ANN_B1 struggled to capture complex relationships, resulting in higher errors and poorer accuracy.

Optimal performance was achieved by ANN_A4 for polar angle prediction and ANN_B2 for refractive index prediction when using the full dataset with TrainLM. The K-fold results for both tasks, however, illustrate the limitations posed by smaller effective training sets and the sensitivity of certain algorithms to initialization and optimization. For refractive index, the augmented dataset and min–max normalization enabled K-fold networks like ANN_B4* to generalize effectively, even if the nominal R^2^ was slightly lower than the full-dataset TrainLM network. These findings emphasize that architectural tuning is crucial for efficient and accurate predictions in LC systems, while model complexity must align with the size and quality of the training dataset. Overly complex networks risk memorizing noise, especially with limited data, while simpler models may lack the capacity to capture underlying relationships. A robust, representative dataset combined with an appropriately complex architecture is essential for effective modeling of LC behavior.

## 6. Conclusions

This study developed ANNs to predict the mean steady-state polar angle and refractive index in LC systems by leveraging surface viscosity, anchoring energy, and other input parameters. Using the train, validation, and test method, ANN_A4 (R^2^ = 0.9995) and ANN_B2 (R^2^ = 0.9969) achieved the highest predictive accuracy, demonstrating excellent performance on single splits. However, when evaluated using K-fold cross-validation, the results differed significantly, with the best performance observed for ANN_A5* (R^2^ = 0.40767) and ANN_B4* (R^2^ = 0.93799), highlighting the sensitivity of some architectures to reduced effective training sets and the importance of robust validation strategies to assess generalization.

These results demonstrate that AI-driven approaches can offer significant advantages over traditional numerical methods, which often require extensive computational resources and complex parameter tuning. The ANN’s ability to generalize within the trained parameter space suggests that machine learning can be a powerful tool for modeling LC behavior, potentially accelerating research and innovation in this field. These results emphasize that while AI-driven approaches can model LC behavior efficiently and capture complex nonlinear dependencies, predictive accuracy depends strongly on dataset size, representativeness, and training methodology.

Future studies could explore alternative optimization strategies, regularization techniques, or physics-informed neural networks to improve stability and generalization.

In summary, this work demonstrates that ANNs, combined with systematic validation such as K-fold cross-validation, provide a viable and efficient alternative to traditional LC simulations and offer potential for broader prediction of complex LC physical parameters.

## Figures and Tables

**Table 1:** Architecture of ANN models assessed to predict the mean steady-state polar angle.

Model	Hidden Layers	Neurons per Layer
ANN_A1	3	10
ANN_A2	3	25
ANN_A3	3	50
ANN_A4	5	10
ANN_A5	5	20

**Table 2:** Architecture of ANN models assessed to predict the refractive index.

Model	Hidden Layers	Neurons per Layer
ANN_B1	2	100
ANN_B2	3	10
ANN_B3	3	25
ANN_B4	5	10
ANN_B5	5	20
ANN_B6	5	50

**Table 3:** Training performance of different ANNs to predict the mean steady-state polar angle.

Model	Number of Hidden Layers	Number of Neurons per Layer	R^2^	Epochs	Reason for Termination
ANN_A1	3	10	0.8042	531	Validation criteria met
ANN_A2	3	25	0.9597	1000	Max epochs reached
ANN_A3	3	50	0.6574	489	Manually terminated
ANN_A4	5	10	0.9995	1000	Max epochs reached
ANN_A5	5	20	0.9993	1000	Max epochs reached

**Table 4:** Training performance of ANNs for prediction of mean steady state polar angle with K-Fold cross-validation.

Model	Mean RMSE	Standard RMSE	Mean MAE	Standard MAE	Mean R^2^
ANN_A1*	0.18136	0.00552	0.15089	0.00504	0.3325
ANN_A2*	0.17613	0.00590	0.14562	0.00554	0.37032
ANN_A3*	0.17271	0.00417	0.14290	0.00384	0.39471
ANN_A4*	0.18149	0.00400	0.14900	0.00457	0.33177
ANN_A5*	0.17073	0.00725	0.13974	0.00904	0.40767

* denotes training using TrainSCG.

**Table 5:** Training performance of different ANNs to predict the refractive index.

Model	Number of Hidden Layers	Number of Neurons per Layer	R^2^	Epochs	Reason for Termination
ANN_B1	2	100	0.7751	117	Validation criteria met
ANN_B2	3	10	0.9969	320	Performance criteria met
ANN_B3	3	25	0.9812	287	Performance criteria met
ANN_B4	5	10	0.9209	209	Performance criteria met
ANN_B5	5	20	0.9566	111	Performance criteria met
ANN_B6	5	50	0.9568	159	Validation criteria met

**Table 6:** Training performance of ANNs for prediction of refractive index with K-Fold cross-validation.

Model	Mean RMSE	Standard RMSE	Mean MAE	Standard MAE	Mean R^2^
ANN_B1*	2.0877	0.37926	0.55173	0.047546	0.69954
ANN_B2*	1.3841	0.40119	0.36165	0.066311	0.86613
ANN_B3*	1.5756	0.63294	0.3612	0.079469	0.81132
ANN_B4*	0.91864	0.21799	0.22263	0.038429	0.93799
ANN_B5*	1.1709	0.3593	0.2895	0.069906	0.9003
ANN_B6*	1.996	0.40984	0.42232	0.034549	0.72118

* denotes training using TrainSCG.

## Data Availability

The raw data supporting the conclusions of this article will be made available by the authors on request.

## Abbreviations

The following abbreviations are used in this manuscript:

**Acronyms**	
AI	artificial intelligence
ANN	artificial neural network
DNA	deoxyribonucleic acid
LC	liquid crystal
MC	Monte Carlo
MD	molecular dynamics
MSE	mean squared error
POM	polarizing optical microscope
ReLU	rectified linear unit
**Greek Letter**	
θ	Polar angle
